# Circ‐LAMP1 contributes to the growth and metastasis of cholangiocarcinoma via miR‐556‐5p and miR‐567 mediated YY1 activation

**DOI:** 10.1111/jcmm.16392

**Published:** 2021-03-06

**Authors:** Yi Xu, Ping Gao, Zhidong Wang, Zhilei Su, Guanqun Liao, Yi Han, Yifeng Cui, Yue Yao, Xiangyu Zhong

**Affiliations:** ^1^ Department of Hepatopancreatobiliary Surgery Second Affiliated Hospital of Harbin Medical University Harbin China; ^2^ Young Scholar of General Surgery Climbing Program of China China; ^3^ Department of Endocrinology and Metabolism Second Affiliated Hospital of Harbin Medical University Harbin China; ^4^ Department of Interventional Radiology Stanford University School of Medicine Stanford CA USA; ^5^ Department for Visceral Thoracic and Vascular Surgery at the University Hospital Technical University Dresden Dresden Germany; ^6^ Department of Hepatic Surgery First Affiliated Hospital of Harbin Medical University Harbin China

**Keywords:** cholangiocarcinoma, circ‐LAMP1, circRNA, miR‐556‐5p, miR‐567, YY1

## Abstract

Dysregulation of circular RNAs (circRNAs) executes important regulatory roles in carcinogenesis. Nonetheless, few studies focused on the mechanisms of circRNAs in cholangiocarcinoma (CCA). qRT‐PCR was applied to verify the dysregulated circRNAs in CCA. Fisher's exact test, Kaplan‐Meier analysis and Cox regression model were utilized to investigate the clinical implications of circ‐LAMP1 in the patients with CCA. The viability, apoptosis, migration and invasion of CCA cells were detected after silencing/overexpression of circ‐LAMP1. Xenograft and lung metastasis assays were performed to verify the in vitro results. The regulatory networks of circ‐LAMP1 were unveiled by bioinformatic analysis, RNA immunoprecipitation (RIP), RNA pulldown and luciferase reporter assays. Up‐regulation of circ‐LAMP1 was found in CCA tissue samples and cell lines. Enhanced level of circ‐LAMP1 was linked to clinical severity, high post‐operative recurrence and poor prognosis for the patients with CCA. Gain/loss‐of‐function assays confirmed the oncogenic role of circ‐LAMP1 in mediating cell growth, apoptosis, migration and invasion. Nevertheless, the level of circ‐LAMP1 had no effect on normal biliary epithelium proliferation and apoptosis. Animal study further verified the in vitro data. Mechanistically, circ‐LAMP1 directly sponged miR‐556‐5p and miR‐567, thereby releasing their suppression on YY1 at post‐transcriptional level. Rescue assay indicated that the oncogenic role of circ‐LAMP1 is partially dependent on its modulation of YY1 in CCA. In summary, this study suggested that circ‐LAMP1 might be used as a promising biomarker/therapeutic target for CCA.

## 1 INTRODUCTION

Cholangiocarcinoma (CCA) is an aggressive malignancy in hepatobiliary system. A considerable portion of patients are diagnosed at advanced stages, which lack effective therapies, leading to poor treatment outcome.^1^ The pathogenesis of CCA is a complex process, which was affected by various factors and oncogenes. Tumour biomarkers are of great clinical value and crucial significance for the early detection, targeted treatment and therapeutic effect evaluation.^2^, ^3^ Therefore, it is enormously crucial to discover novel and reliable treatment strategy for CCA.

Circular RNAs (circRNAs) are a series of ncRNAs with limited protein‐coding capacity.^4^, ^5^ Up to now, lots of circRNAs are discovered from mammalian cells via high throughput sequencing and bioinformatics analysis.^5^ Growing findings imply that circRNAs are tightly implicated in the dysregulation of gene transcription and translation, suggesting that circRNAs may go for the development of human diseases.^6^, ^7^, ^8^ Currently, circRNAs are known to have the following roles: (a) they act as “miRNA sponges” to modulate miRNAs activity^4^; (b) they interact with RNA‐binding proteins^9^; and (c) they are translated to specific proteins.^10^ CircRNAs, especially those with miRNA response elements, bind to miRNAs, thereby acting as competing endogenous RNAs (ceRNAs).^11^ For instance, ciRS‐7 reduces miR‐7 activity and thus increases several oncogenic factors expression.^12^, ^13^ The evidence indicates that the circRNA‐miRNA‐mRNA axis is essential to the mechanisms that determine the progress of cancers.

The current study identifies a novel CCA‐related circRNA, circ‐LAMP1, which is elevated in CCA tissues analysed by circRNA microarray. Circ‐LAMP1 (hsa_circRNA_101303; circBase ID: hsa_circ_0030998; circBank ID: hsa_circLAMP1_010) is located on chr13:113963957‐113964177. The spliced sequence length of circ‐LAMP1 is 220 nt. The genomic structure illustrates that circ‐LAMP1 is looped by the exon‐3 of LAMP1 gene. In the study, up‐regulated circ‐LAMP1 correlates with the clinical severity and prognosis of CCA patients. In vitro and in vivo assays revealed its effect in promoting the growth and metastasis of CCA cells. Mechanistically, we identified that circ‐LAMP1 sponges miR‐556‐5p and miR‐567 to up‐regulate YY1 at post‐transcriptional level. Furthermore, we demonstrated that circ‐LAMP1 exerts oncogenic properties via miR‐556‐5p/miR‐567/YY1 axis.

## 2 MATERIALS AND METHODS

### 2.1 Clinical samples

A total of 216 individuals were enrolled from the Second Affiliated Hospital of Harbin Medical University. The use of patient specimens was authorized by the Ethics Committee of our hospital. Each patient signed the written informed consent prior to sample donation. None of the patients received pre‐operative anti‐tumour treatment before specimen collection. Immediately after surgical resection, the specimens were stored at −80°C.

### 2.2 Cell lines and culture

Human CCA cells (HCCC‐9810 and RBE) were acquired from Chinese Academy of Sciences (Shanghai). Huh‐28, QBC939, HuCCT1, CCLP1 and the normal cell line (HIBEC) were acquired from Professor Lianxin Liu (Changjiang Scholar, University of Science and Technology of China) as a gift. The cells were maintained in complete medium containing 90% RPMI‐1640 (Hyclone) and 10% foetal bovine serum (FBS; Gibco). All the cells were placed in a 5% CO_2_ humidity condition at 37°C. The solution was changed every 3 days cell passage. Cells in all of the following experiments were taken in the logarithmic growth phase. All cell lines were passaged for no more than 6 months.

### 2.3 qRT‐PCR and cell transfection

Total RNA from tissues and cells was isolated by utilizing TRIzol in accordance with manufacturer's descriptions. First Strand cDNA Synthesis Kit (Roche) was recruited to generate cDNA from harvested RNA. Based on this, the equal cDNA was mixed with RNase free water, primers and reagents of SYBR‐Green PCR kit (QIAGEN). Relative gene expression was calculated by the 2^−ΔΔCt^ method. The expression of YY1 mRNA and circ‐LAMP1 was standardized to GAPDH. In addition, miRNAs (miR‐556‐5p, miR‐567 and miR‐615‐5p) expression was standardized to U6. The primer sequences are shown: circ‐LAMP1 (Forward: 5’‐ CGTCCAGCTCATGAGTTTTGT −3’, Reverse: 5’‐ AGACTGGGGTCAGAAGTGTTC −3’); GAPDH (Forward: 5’‐ GGGAGCCAAAAGGGTCAT −3’, Reverse: 5’‐ GAGTCCTTCCACGATACCAA −3’). U6 (Forward: 5’‐ ATTGGAACGATACAGAGAAGATT −3’, Reverse: 5’‐ GGAACGCTTCACGAATTTG −3’).

The specific siRNAs targeting circ‐LAMP1/YY1 and si‐NC were obtained from Ribio. Sh‐circ‐LAMP1‐1, circ‐LAMP1 vector, miR‐556‐5p/567/NC mimics and inhibitor were constructed by GenePharma. 2 × 10^5^ CCA cells per well were plated in 2.5 cm dishes and then transfected with siRNAs or the scrambled oligo nucleotides (50 nM) using Lipofectamine 3000 following the manufacture instructions. The targeted sequences of si/shRNA‐circ‐LAMP1 are listed below: si/sh‐circ‐LAMP1‐1, 5'‐ AGCTCCAAAGAACATGACCTT −3' and si/sh‐circ‐LAMP1‐2, 5'‐ TGCGAGCTCCAAAGAACATGA −3'.

### 2.4 Cell counting kit‐8 (CCK‐8) and colony formation assays

Cell viability curves were measured by CCK‐8 experiment. KMBC or RBE cells were seeded in 96‐well dishes and then incubated at 37°C for 2 h. Afterwards, 10 μl of CCK‐8 reagent (Beyotime) was supplied and the cells were incubated at 37°C for 2 h in a cell incubator. Finally, the absorbance was detected at 450 nm.

For clonogenic assay, five hundred transfected cells were placed into 2.5 cm dishes and maintained at 37°C. After 10 days, visible colonies had formed. The colonies were fixed with paraformaldehyde, stained by crystal violet and photographed.

### 2.5 Cell apoptosis determination

Cells at logarithmic growth phase were harvested by trypsin. After resuspend in 400 μl of 1× binding buffer, the cells were then stained by 5 μl of FITC‐Annexin V and 5 μl of PI (Beyotime, Haimen, China). A flow cytometer was utilized to evaluate apoptotic cells (BD Biosciences).

### 2.6 Wound healing assay

KMBC and RBE cells were planted in 6‐well dishes overnight. The cell monolayer was wounded with a pipette tip to create a scratch. Afterwards, the cells were washed with PBS and the serum‐free medium was added. Images were captured at 24/36 h following the initial scratch to evaluate cell migration rate.

### 2.7 Transwell experiments

After being dispersed with 0.25% trypsin, the CCA cells (1 × 10^5^ for cell invasion and 5 × 10^4^ for cell migration) were centrifuged, resuspended and dispersed in the top compartment of transwell unit (Corning, Beijing, China). Matrigel (BD Biosciences) was used in invasion experiment, but not in migration experiment. At the same time, the RPMI‐1640 containing 10% FBS was supplied into the lower compartment. After culturing for 36 h, the cells in the top membranes were abandoned, and the cells in the lower surface of the upper chamber were fixed and stained. Lastly, the migrated/invaded cells were visualized and counted microscopically.

### 2.8 Dual‐luciferase reporter gene test

The wild‐type (circ‐LAMP1 wt) or mutant‐type (circ‐LAMP1 mut) containing the putative binding site of miR‐556‐5p or miR‐567 was amplified and cloned into the pmirGLO‐control luciferase reporter vectors (Promega Corporation). Target sequence and mutation sequence were constructed according to potential binding sites of miR‐556‐5p or miR‐567 on YY1 3’‐UTR (YY1 3’‐UTR wt and YY1 3’‐UTR mut). CCA cells were co‐transfected with the reporter plasmid and miR‐556‐5p mimics, miR‐567 mimics or mimics‐NC by Lipofectamine 3000. After 36 h, the luciferase activity was analysed by a dual‐luciferase reporter assay system.

### 2.9 RNA Immunoprecipitation (RIP) assay

RIP was carried out using the Magna RIP RNA‐Binding Protein Immunoprecipitation Kit (Millipore) in agreement with the manufacturer's instruction. KMBC and RBE cell lysates were obtained and incubated with RIP buffer containing magnetic beads conjugated with human anti‐Ago2 antibody or normal mouse IgG (control). RNA was extracted from immunoprecipitate and analysed by qRT‐PCR.

### 2.10 RNA pulldown assay

The biotin‐labelled circ‐LAMP1 probe targeting the junction sequence of circ‐LAMP1 was in vitro synthesized by Genecreate (Wuhan, China), followed by incubation with cell lysate at 4°C overnight. Then, the above complex was incubated with streptavidin‐conjugated magnetic beads at room temperature for 2 h. Lastly, interacted RNAs were purified and evaluated by qRT‐PCR.

### 2.11 Western blotting

Immunoblotting assay was performed as per previously described protocols.^7^ Anti‐YY1 (ab109237, 1:5000) and anti‐GAPDH (ab16891, 1:10,000) were acquired from Abcam (Cambridge).

### 2.12 In vivo*experiments*

The animal assay was authorized by the Institutional Animal Care and Use Committee of our hospital. KMBC cells (6 × 10^6^) were injected into the left flank of nude mice (n = 4/group, six‐week‐old) subcutaneously. The volume of the formed tumour was measured every 3 days at 6 days post‐injection. Tumour volume was calculated following the formula: volume = length × width^2^ × 0.5. After injection for 27 days, the mice were killed, and the subcutaneous xenografts were harvested and weighed.

For cell metastasis detection, the transfected KMBC cells (2 × 10^6^) were injected into the tail vein to construct the lung metastatic model (n = 4/group). Four week later, the mice were killed and their lungs were harvested and subjected to H&E staining.

### 2.13 Bioinformatics analyses

Circular RNA Interactome (https://circinteractome.nia.nih.gov) and circBank (http://www.circbank.cn) were applied to predict the target miRNAs of circ‐LAMP1. The target genes of miR‐556‐5p and miR‐567 were predicted using the target gene prediction software, TargetScan (http://www.targetscan.org/).

### 2.14 Data analysis

Data analyses of the project were accomplished by SPSS 22.0 software (IBM). For group comparison, the differences of the two‐group and multiple‐groups were analysed via Student's *t* test and one‐way analysis of variance with Tukey's test, respectively. Correlation test was carried out to examine the correlation between miR‐556‐5p/miR‐567, circ‐LAMP1 and YY1. Survival curves were estimated applying Kaplan‐Meier method with log‐rank test. Multivariate cox regression analysis was used to determine the independent factors that affect overall survival (OS) and disease‐free survival (DFS) for CCA patients. *P* <.05 meant the existence of statistical significance.

## 3 RESULTS

### 3.1 Circ‐LAMP1 is up‐regulated in CCA and correlates with unfavourable prognosis

**FIGURE 1:**
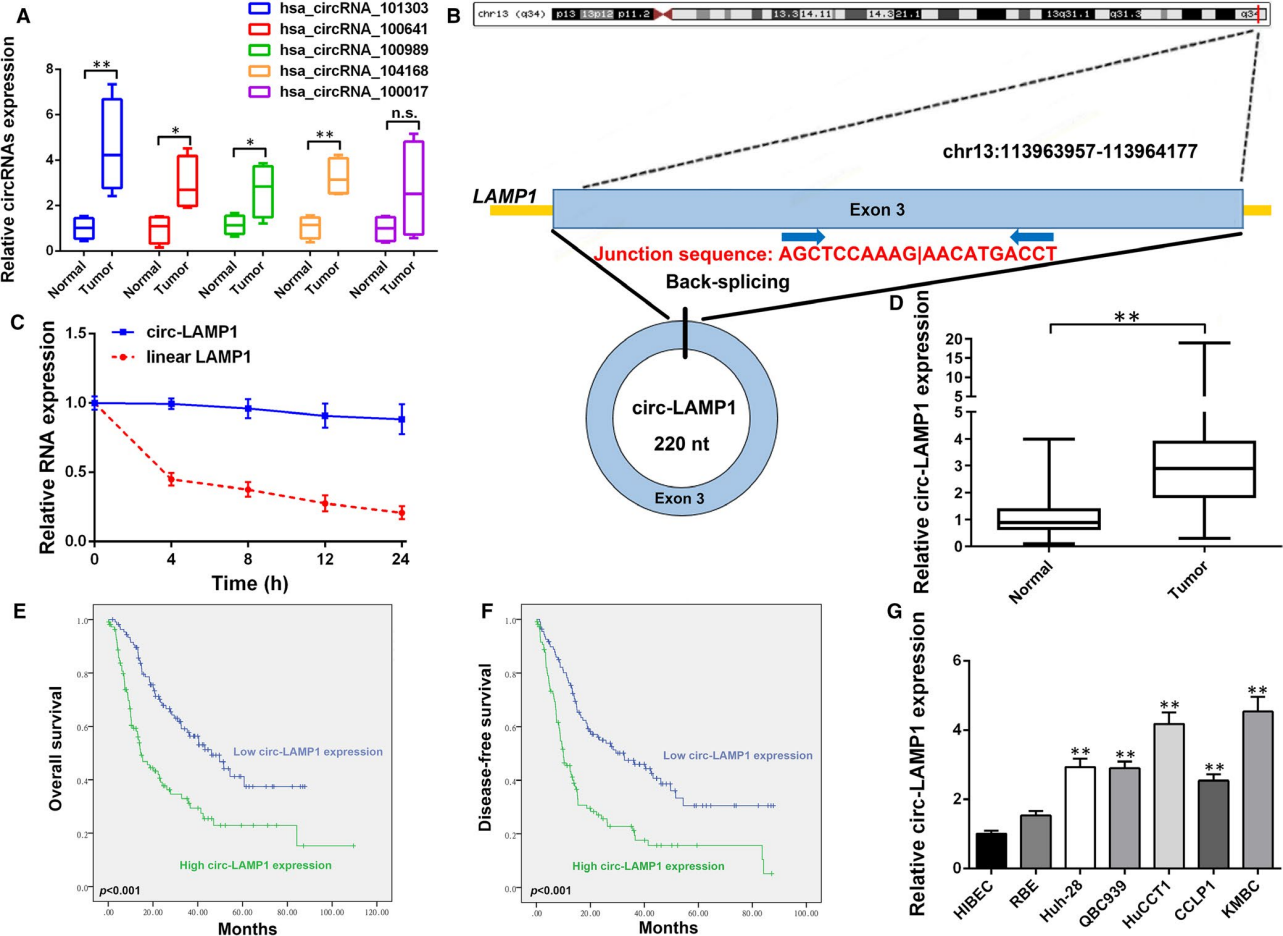
Relative expression of circ‐LAMP1 in CCA tissues and cells and its clinical significance. A, Relative expression of five circRNAs in 15 pairs of CCA tissue samples and adjacent non‐cancerous tissues measured by qRT‐PCR. B, Schematic representation of circ‐LAMP1 formation. C, Relative circ‐LAMP1 and linear LAMP1 mRNA expression at different time point. D, qRT‐PCR for circ‐LAMP1 expression in cancerous/normal tissues. E, Kaplan‐Meier analysis of OS in CCA patients according to circ‐LAMP1 expression. F, Kaplan‐Meier analysis of DFS in CCA patients according to circ‐LAMP1 expression. G, qRT‐PCR for circ‐LAMP1 expression in CCA cells and HIBEC. *P <.05, ** *P* <.01

We previously performed circRNA microarray by using four pairs of CCA tissues and adjacent normal samples. Among the 224 differentially expressed circRNAs (fold change > 2, *P* < .05), 97 were up‐regulated, and 127 were down‐regulated in CCA tissues in comparison with those in normal tissues. We randomly selected five elevated circRNAs (hsa_circRNA_101303, hsa_circRNA_100641, hsa_circRNA_100989, hsa_circRNA_104168 and hsa_circRNA_100017) for further study. As a result, hsa_circRNA_101303 was the most up‐regulated one among the recruited circRNAs (Figure 1A). Hsa_circRNA_101303 originates from exon‐3 of a protein‐coding gene locus, *LAMP1* (Figure 1B). In addition, the half‐life of circular form of LAMP1 (circ‐LAMP1) is remarkably longer than that of linear LAMP1 mRNA (Figure 1C). To explore the expression status of circ‐LAMP1, we evaluated the level of circ‐LAMP1 in 216 pairs of CCA tissues compared with that of normal specimens. qRT‐PCR analysis convinced that circ‐LAMP1 was apparently augmented in CCA tissues (Figure 1D). The recruited patients were divided into two groups followed by the median value of circ‐LAMP1 expression. Notably, circ‐LAMP1 in cancerous tissues was linked to number of tumours (*P* =.003) and TNM stages (*P* =.013), both significantly (Supplementary file 1, Table S1). Kaplan‐Meier analysis appraised possible significance for circ‐LAMP1 degree in predicting OS and DFS for CCA cases. In accordance with the relevant findings, CCA cases harbouring high circ‐LAMP1 degrees experienced a worse OS than low ones (*P* < .001, Figure 1E). Additionally, circ‐LAMP1 expression in CCA tissue samples was remarkably linked to high post‐operative recurrence for the patients with CCA (*P* < .001, Figure 1F). Multivariate analysis evaluated whether circ‐LAMP1 expression level or other clinicopathological characteristics were independent prognostic markers for CCA cases. Consequently, high circ‐LAMP1 level is a valuable indicator for predicting OS (*P* = .001, Supplementary file 2, Table S2). Furthermore, higher expression of circ‐LAMP1 was tightly correlated with patients’ DFS (*P* = .001, Supplementary file 3, Table S3). Circ‐LAMP1 expression in six CCA cells and HIBEC was further investigated. As exhibited in Figure 1G, circ‐LAMP1 was overexpressed in almost all the recruited CCA cells than HIBEC.

### 3.2 Circ‐LAMP1 accelerates CCA cell progression in vitro

**FIGURE 2:**
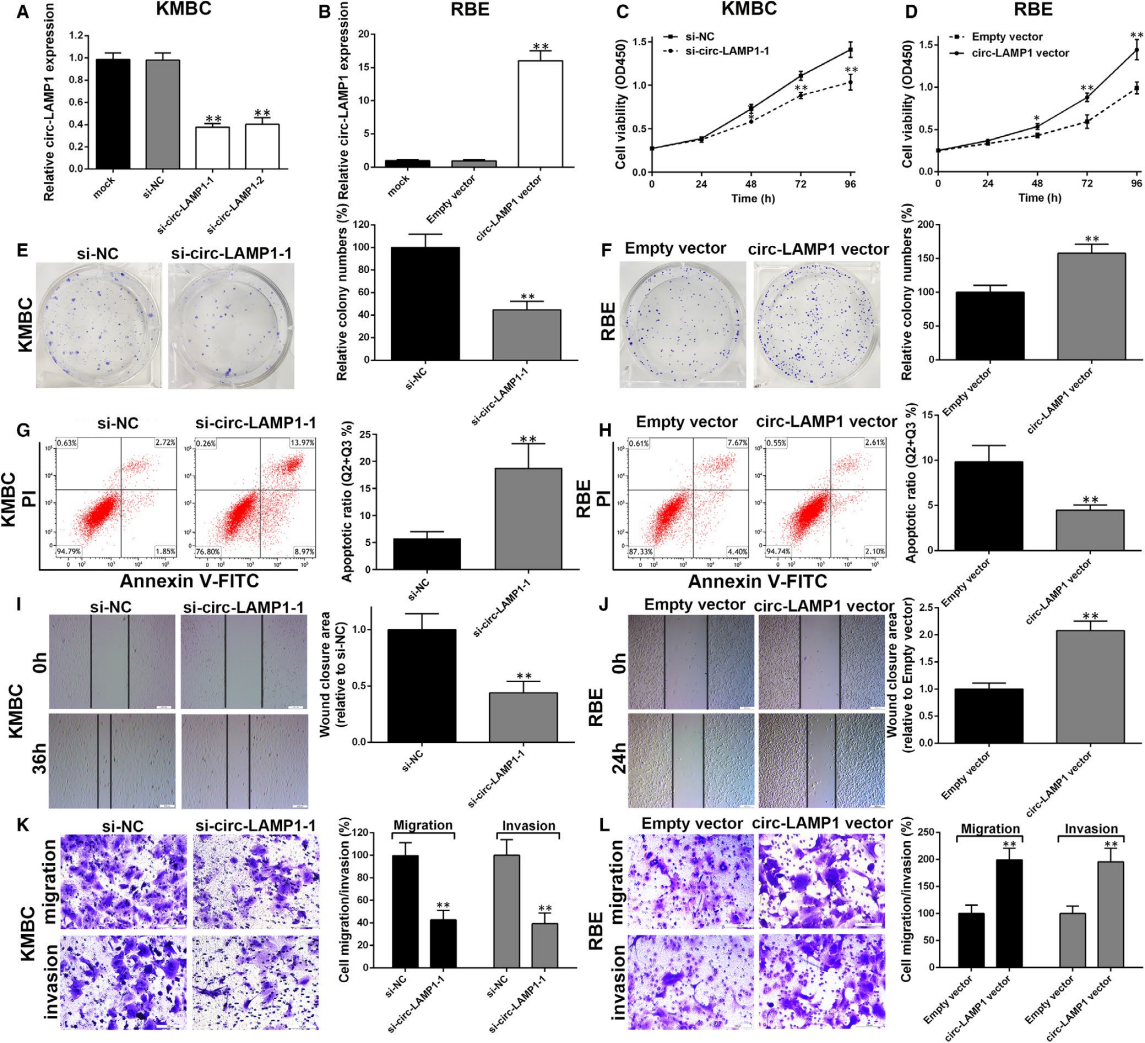
Circ‐LAMP1 promotes CCA cell progression in vitro. A, qRT‐PCR for circ‐LAMP1 expression after silencing of circ‐LAMP1 in KMBC cells. B, qRT‐PCR for circ‐LAMP1 expression after up‐regulation of circ‐LAMP1 in RBE cells. C, Cell viability was detected by CCK‐8 after silencing of circ‐LAMP1 in KMBC cells. D, Cell viability was detected by CCK‐8 after up‐regulation of circ‐LAMP1 in RBE cells. E, Clone forming capacity was detected by colony formation assay after silencing of circ‐LAMP1 in KMBC cells. F, Clone forming capacity was detected by colony formation assay after up‐regulation of circ‐LAMP1 in RBE cells. G, Cell apoptosis was detected by flow cytometric assay after silencing of circ‐LAMP1 in KMBC cells. H, Cell apoptosis was detected by flow cytometric assay after up‐regulation of circ‐LAMP1 in RBE cells. I, Cell migration was detected by wound healing assay after silencing of circ‐LAMP1 in KMBC cells; Scale bars = 200 μm. J, Cell migration was detected by wound healing assay after up‐regulation of circ‐LAMP1 in RBE cells; Scale bars = 200 μm. K, Cell migration and invasion was detected by transwell assay after silencing of circ‐LAMP1 in KMBC cells; Scale bars = 100 μm. L, Cell migration and invasion was detected by transwell assay after up‐regulation of circ‐LAMP1 in RBE cells; Scale bars = 100 μm. *P < .05, **P < .01

The results described above indicate that circ‐LAMP1 was elevated in CCA and thus may be an oncogene. Hence, we determined whether circ‐LAMP1 could regulate cell progression in vitro. KMBC cells (with highest circ‐LAMP1 expression) was utilized to perform knockdown study, and the knockdown efficiency was confirmed by qRT‐PCR. As Figure 2A exhibited, both of the siRNAs targeted to the spliced junction of circ‐LAMP1 were effective. Si‐circ‐LAMP1‐1 was utilized for further knockdown study for the reason that it has a better knockdown efficiency. Additionally, RBE cell line (with lowest circ‐LAMP1 expression) was selected for gain‐of‐function assay and the overexpression efficiency was favourable (Figure 2B). CCK‐8 and clone formation assays were applied to examine the effect of silenced/overexpressed circ‐LAMP1 on the proliferation of CCA cells. The data illustrated decreased cell viability/clonogenic capacity in circ‐LAMP1‐underexpressing KMBC cells (Figure 2C,E). Conversely, ectopic expressed circ‐LAMP1 enhanced RBE cell growth and the number of colonies (Figure 2D, F). Furthermore, flow cytometry was conducted to determine whether circ‐LAMP1 regulated CCA cell proliferation at the level of cell apoptosis process. The results showed more apoptotic cells in circ‐LAMP1 depletion group relative to the control group (Figure 2G). Moreover, apoptotic cells were found to be remarkably decreased in circ‐LAMP1 up‐regulated cells (Figure 2H). Cell migration of CCA cells were detected after circ‐LAMP1 expression was knocked down/overexpressed. The data of wound healing and transwell migration tests verified that cell migration of KMBC cells was inhibited by transfected with si‐circ‐LAMP1‐1 (Figure 2I). Elevated circ‐LAMP1 induced the opposite effect in RBE cells (Figure 2J). To explore whether circ‐LAMP1 affects cell metastatic properties, Transwell invasion assay was conducted. As a result, silenced circ‐LAMP1 induced down‐regulation of cell invasive potential (Figure 2K). By contrast, elevated circ‐LAMP1 enhanced cell invasion in RBE cells (Figure 2L).

### 3.3 Circ‐LAMP1 contributes to CCA progression by sponging miR‐556‐5p and miR‐567

**FIGURE 3:**
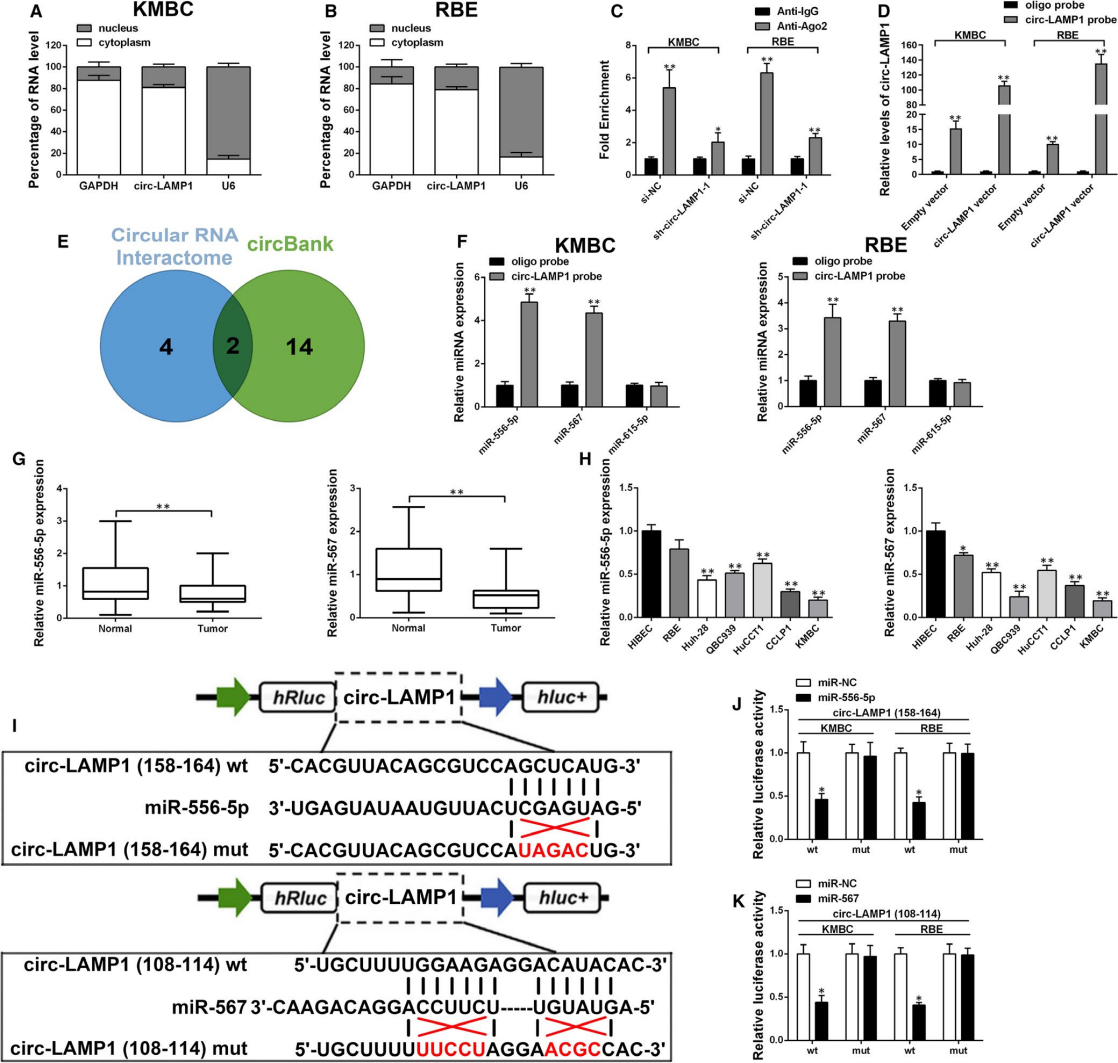
Circ‐LAMP1 directly sponges miR‐556‐5p and miR‐567 in CCA cells. A, B, qRT‐PCR detection of the percentage of circ‐LAMP1 in the cytoplasmic and nuclear fractions of KMBC and RBE cells. C, Ago2‐RNA RIP assay for circ‐LAMP1 levels in KMBC and RBE cells after transfection. D, Lysates prepared from KMBC and RBE cells after transfection were subjected to RNA pulldown assay. E, Venn diagram showing the number of overlapping miRNAs. F, qRT‐PCR for miR‐556‐5p, miR‐567 and miR‐615‐5p expression in KMBC and RBE lysates. G, MiR‐556‐5p and miR‐567 expression in CCA/normal tissues analysed by qRT‐PCR. H, MiR‐556‐5p and miR‐567 expression in HIBEC and CCA cell lines analysed by qRT‐PCR. I, Schematic illustration of circ‐LAMP1‐wt and circ‐LAMP1‐mut luciferase reporter vectors. J, K, The binding ability between circ‐LAMP1 and miR‐556‐5p/567 was measured by dual‐luciferase reporter assay in KMBC and RBE cells. *P < .05, **P < .01

After isolation of nuclear and cytoplasmic fractions, qRT‐PCR results indicated the predominant cytoplasmic distribution of circ‐LAMP1 (Figure 3A, B). We then asked whether circ‐LAMP1 may interact with certain miRNAs in CCA cells. We found that circ‐LAMP1 was obviously enriched in anti‐Ago2 than that in anti‐IgG and was less enriched after circ‐LAMP1 silenced (Figure 3C). A biotin‐labelled circ‐LAMP1 probe was applied to pull down circ‐LAMP1. As Figure 3D exhibited, circ‐LAMP1 up‐regulation remarkably enhanced the pulldown efficiency. After getting intersection of two bioinformatic prediction databases (Circular RNA Interactome and circBank), miR‐556‐5p and miR‐567 were identified as the common miRNAs that may be sponged by circ‐LAMP1 (Figure 3E). A previous study indicated that circ‐LAMP1 could interact with miR‐615‐5p in T‐cell lymphoblastic lymphoma.^14^ Therefore, miR‐615‐5p was also included in the subsequent study. We then isolated miRNAs after pulldown assay and measured the 3 predicted miRNAs expression by qRT‐PCR. As a result, only miR‐556‐5p and miR‐567 were enriched in RNAs pulled down by the circ‐LAMP1 probe in KMBC and RBE cells (Figure 3F). qRT‐PCR analysis uncovered that miR‐556‐5p and miR‐567 expression was decreased in cancerous tissues relative to normal counterparts (Figure 3G). Furthermore, a majority of CCA cell lines harboured a decreased miR‐556‐5p and miR‐567 expression levels, especially in KMBC cells (Figure 3H). To confirm whether circ‐LAMP1 could directly bind to miR‐556‐5p or miR‐567, dual‐luciferase reporter vectors, which contained the putative binding sites or mutant binding sites for miR‐556‐5p/567, were established (Figure 3I). The results showed that miR‐556‐5p/567 mimics remarkably suppressed the luciferase intensity in wild‐type circ‐LAMP1 reporter gene, but not the mutant‐type (Figure 3J, K).

**FIGURE 4:**
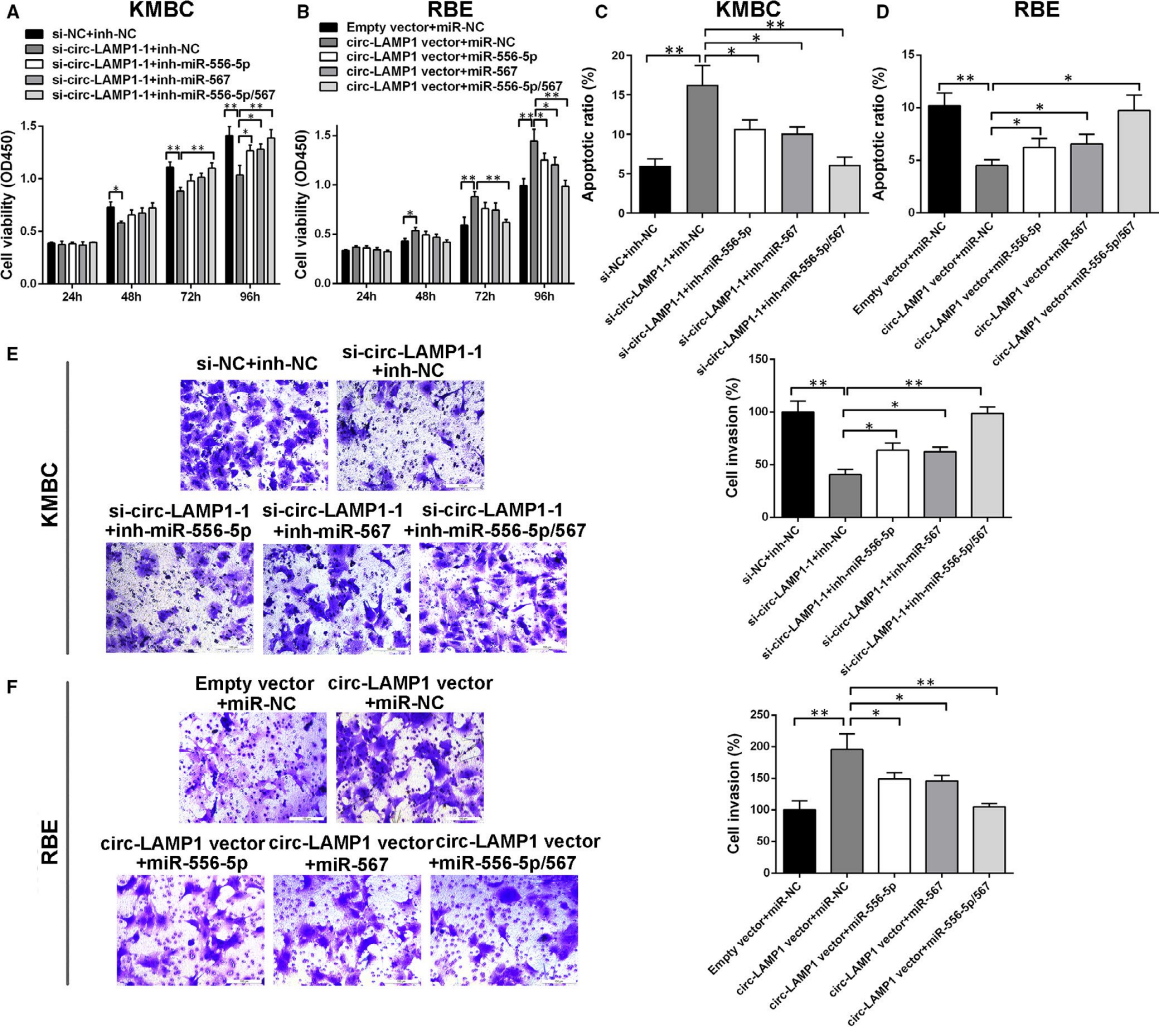
The oncogenic role of circ‐LAMP1 is partly dependent on its regulation of miR‐556‐5p and miR‐567. A, Inhibition of miR‐556‐5p and/or miR‐567 rescued cell viability in circ‐LAMP1‐down‐regulated KMBC cells. B, Ectopic expression of miR‐556‐5p and/or miR‐567 reversed cell viability in circ‐LAMP1‐up‐regulated RBE cells. C, Inhibition of miR‐556‐5p and/or miR‐567 rescued cell apoptosis in circ‐LAMP1‐down‐regulated KMBC cells. D, Ectopic expression of miR‐556‐5p and/or miR‐567 reversed cell apoptosis in circ‐LAMP1‐up‐regulated RBE cells. E, Inhibition of miR‐556‐5p and/or miR‐567 rescued cell invasion in circ‐LAMP1‐down‐regulated KMBC cells. F, Ectopic expression of miR‐556‐5p and/or miR‐567 reversed cell invasion in circ‐LAMP1‐up‐regulated RBE cells. *P < .05, **P < .01

To evaluate the role of miR‐556‐5p and miR‐567 in mediating circ‐LAMP1 induced oncogenic function, rescue assay was conducted. As Figure 4A exhibited, cell proliferation was markedly attenuated after circ‐LAMP1 knockdown. Nonetheless, this suppression was partially reversed by co‐transfected with inh‐miR‐556‐5p or inh‐miR‐567. Furthermore, cell viability was almost totally reversed after co‐silencing of circ‐LAMP1, miR‐556‐5p and miR‐567. Ectopic expressed circ‐LAMP1 caused the growth‐promoting effect in RBE cells. Although after co‐transfected with miR‐556‐5p or miR‐567 mimics, this effect was partially rescued. In the cells co‐transfected with circ‐LAMP1 vector, miR‐556‐5p mimics and miR‐567 mimics, cell viability was almost equal to the cells co‐transfected with empty vector and miR‐NC (Figure 4B). For cell apoptosis and invasion assay, knockdown of miR‐556‐5p or miR‐567 evidently rescued the tumour suppressor function caused by si‐circ‐LAMP1‐1 in KMBC cells. Moreover, after co‐silencing of circ‐LAMP1, miR‐556‐5p and miR‐567, the oncogenic function (cell apoptosis and invasion) of KMBC cells was further recovered (Figure 4C, E). For RBE cells, either co‐transfected with miR‐556‐5p or miR‐567 mimics evidently reversed the oncogenic role induced by circ‐LAMP1 vector. Moreover, enhancement of circ‐LAMP1, miR‐556‐5p and miR‐567 altogether further inhibited cell malignant behaviours (Figure 4D, F).

### 3.4 ***Circ‐LAMP1 up*** *‐* ***regulates YY1 expression via targeting miR‐556‐5p and miR‐567, thereby facilitating CCA progression***

**FIGURE 5:**
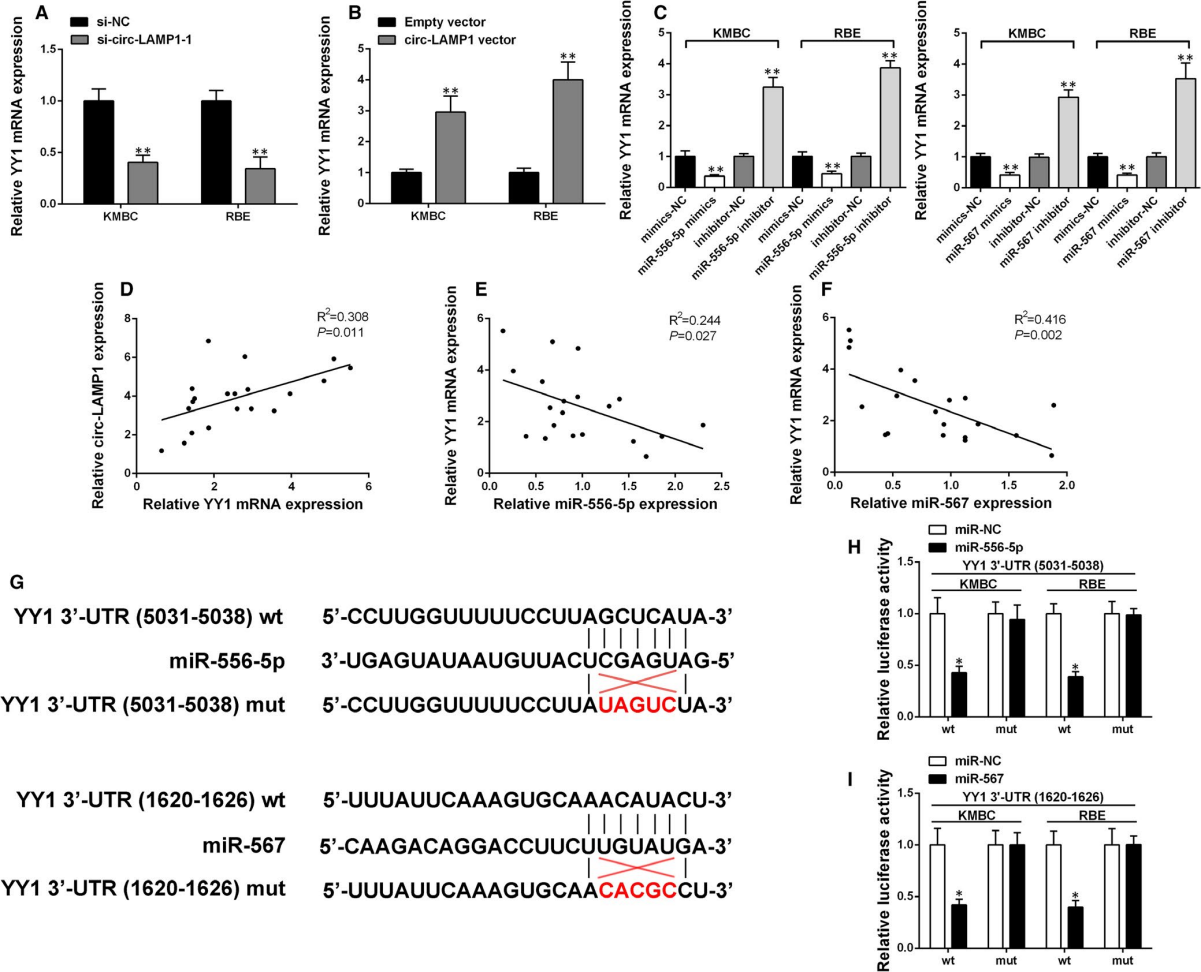
Circ‐LAMP1 elevated YY1 expression by sponging miR‐556‐5p and miR‐567. A, Relative YY1 mRNA expression was determined after silencing of circ‐LAMP1 in KMBC and RBE cells by qRT‐PCR. B, Relative YY1 mRNA expression was determined after up‐regulation of circ‐LAMP1 in KMBC and RBE cells by qRT‐PCR. C, Relative YY1 mRNA expression was determined after silencing/up‐regulation of miR‐556‐5p/567 in KMBC and RBE cells by qRT‐PCR. D, Pearson's correlation analysis of circ‐LAMP1 and YY1 expression in 20 pairs of CCA tissues. X‐axis: Relative YY1 mRNA expression; Y‐axis: Relative circ‐LAMP1 expression. E, Pearson's correlation analysis of YY1 and miR‐556‐5p expression in 20 pairs of CCA tissues. X‐axis: Relative miR‐556‐5p expression; Y‐axis: Relative YY1 mRNA expression. F, Pearson's correlation analysis of YY1 and miR‐567 expression in 20 pairs of CCA tissues. X‐axis: Relative miR‐567 expression; Y‐axis: Relative YY1 mRNA expression. G, Schematic illustration of YY1 3’‐UTR‐wt and YY1 3’‐UTR‐mut luciferase reporter vectors. H, I, The binding ability between YY1 3’‐UTR and miR‐556‐5p/567 was measured by dual‐luciferase reporter assay in KMBC and RBE cells. *P < .05, **P < .01

Based on the bioinformatic prediction database, Targetscan, we found that miR‐556‐5p and miR‐567 may modulate YY1 expression at post‐transcriptional level and, thus, chose YY1 for further study. We first identified that knockdown of circ‐LAMP1 attenuated YY1 expression in KMBC and RBE cell lines (Figure 5A). Conversely, YY1 expression increased after transfection with circ‐LAMP1 vector in both cells (Figure 5B). Transfected with miR‐556‐5p/567 mimics could decrease YY1 expression in CCA cells. Down‐regulation of miR‐556‐5p/567 up‐regulated YY1 mRNA level (Figure 5C). Moreover, YY1 mRNA expression was positively associated with circ‐LAMP1 expression in 20 pairs of CCA specimens as revealed by qRT‐PCR (Figure 5D). Further analysis showed a converse correlation between circ‐LAMP1 and miR‐556‐5p/567 expression (Figure 5E, F). Dual‐luciferase reporter gene test presented that miR‐556‐5p/567 mimics statistically weakened the luciferase activity driven by YY1 3’‐UTR wild‐type (wt), but had no significant alteration in the luciferase activity of the mutant (mut) reporters in KMBC and RBE cells (Figure 5G–I).

**FIGURE 6:**
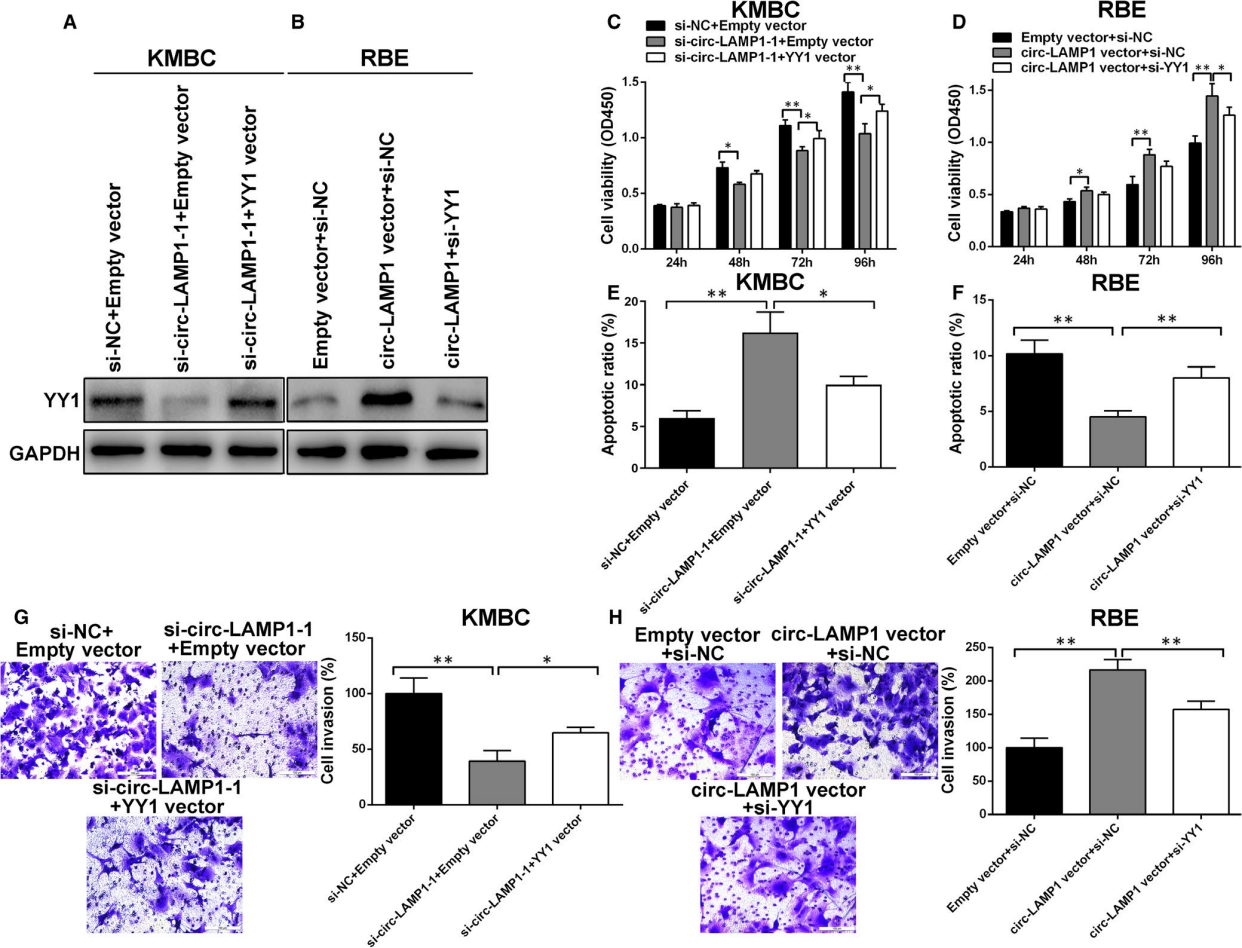
Circ‐LAMP1 regulates CCA cell growth, apoptosis and invasion via up‐regulating YY1 expression. A, B, The protein level of YY1 was measured by Western blotting after transfection in KMBC and RBE cells. C, D, CCK‐8 assay was performed to analyse the proliferation of KMBC and RBE cells after transfection. E, F, Flow cytometric assay was performed to detect the apoptosis of KMBC and RBE cells after transfection. G, H, Transwell assay was performed to detect the invasion of KMBC and RBE cells after transfection. *P < .05, **P < .01

Afterwards, experiments were performed to test whether circ‐LAMP1 acted as an oncogene by elevating YY1 expression in CCA cells. We observed that circ‐LAMP1 down‐regulation decreased YY1 expression level in KMBC cells. Co‐transfected with YY1 vector abolished the effect of circ‐LAMP1 down‐regulation on the YY1 expression (Figure 6A). Additionally, the up‐regulation of YY1 by circ‐LAMP1 vector could be rescued by si‐YY1 in RBE cells (Figure 6B). Functionally, YY1 overexpression partly eliminated the effect of circ‐LAMP1 down‐regulation on cell growth, apoptosis and invasion (Figure 6C, E, G). Furthermore, co‐transfected with circ‐LAMP1 vector and si‐YY1 in RBE cells partially reversed the oncogenic properties caused by circ‐LAMP1 vector (Figure 6D, F, H).

### 3.5 Circ‐LAMP1 can not affect HIBEC proliferation and apoptosis

**FIGURE 7:**
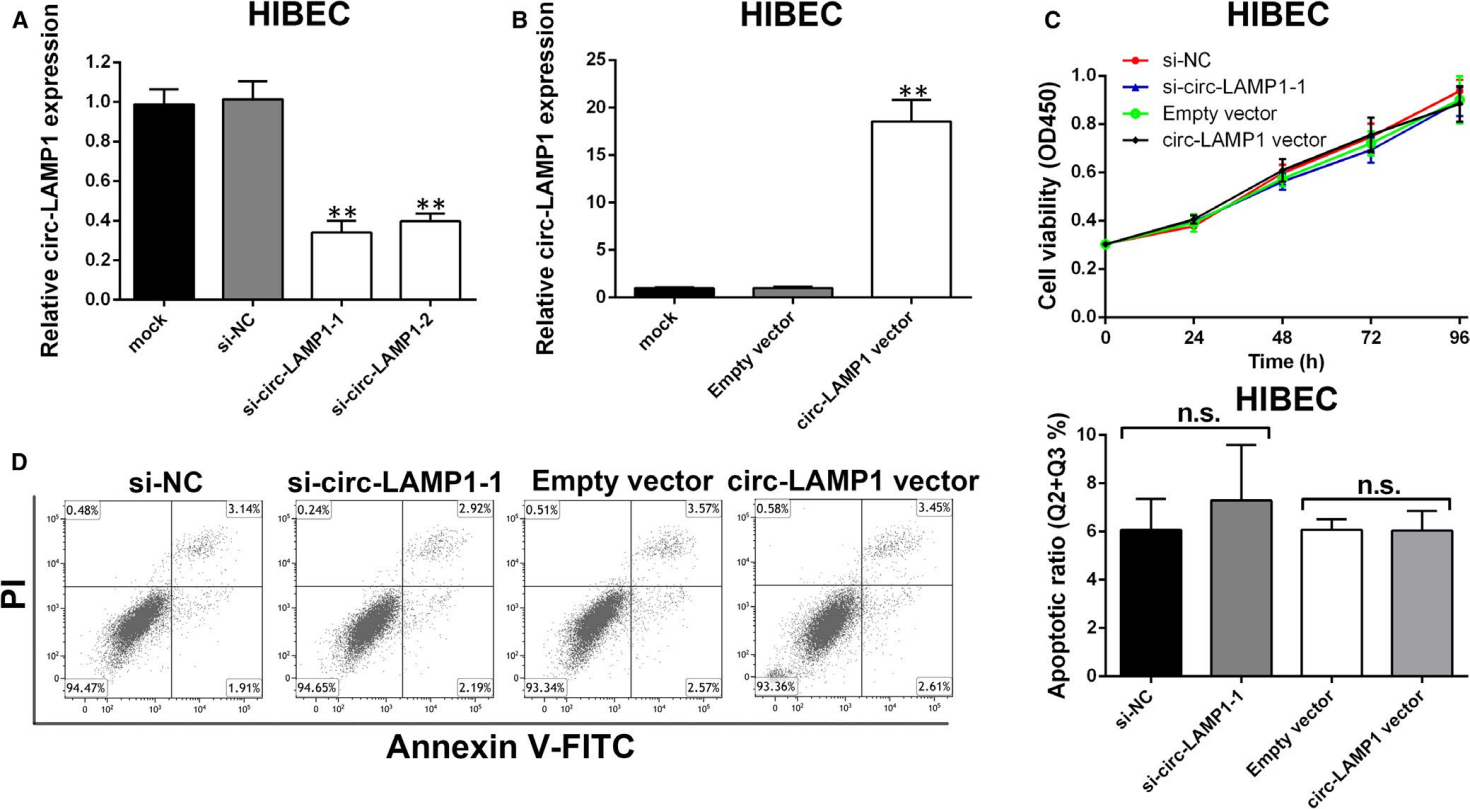
Circ‐LAMP1 expression does not affect HIBEC cell proliferation and apoptosis. A, HIBEC cells were transfected with siRNAs, and siRNA‐depletion efficiency was detected by qRT‐PCR. B, HIBEC cells were transfected with vectors, and circ‐LAMP1‐overexpression efficiency was detected by qRT‐PCR. C, The proliferation of HIBEC cells after transfection was detected by CCK‐8 assays. D, The apoptosis of HIBEC cells after transfection was detected by flow cytometric analysis. **P < .01

To investigate whether circ‐LAMP1 may have side effect on normal biliary epithelium, we knocked down and overexpressed circ‐LAMP1 expression in HIBEC. As Figure 7A demonstrated, the two selected siRNAs had effective knockdown efficiencies. Then, ectopic expression of circ‐LAMP1 in HIBEC was also achieved (Figure 7B). For the part of functional assay, cell viability was not altered after circ‐LAMP1 knockdown/overexpression (Figure 7C). Furthermore, cell apoptotic rate could not be affected by circ‐LAMP1 in HIBEC cells (Figure 7D).

### 3.6 Circ‐LAMP1 deletion resulted in the decrease of tumour growth and metastasis in vivo

**FIGURE 8:**
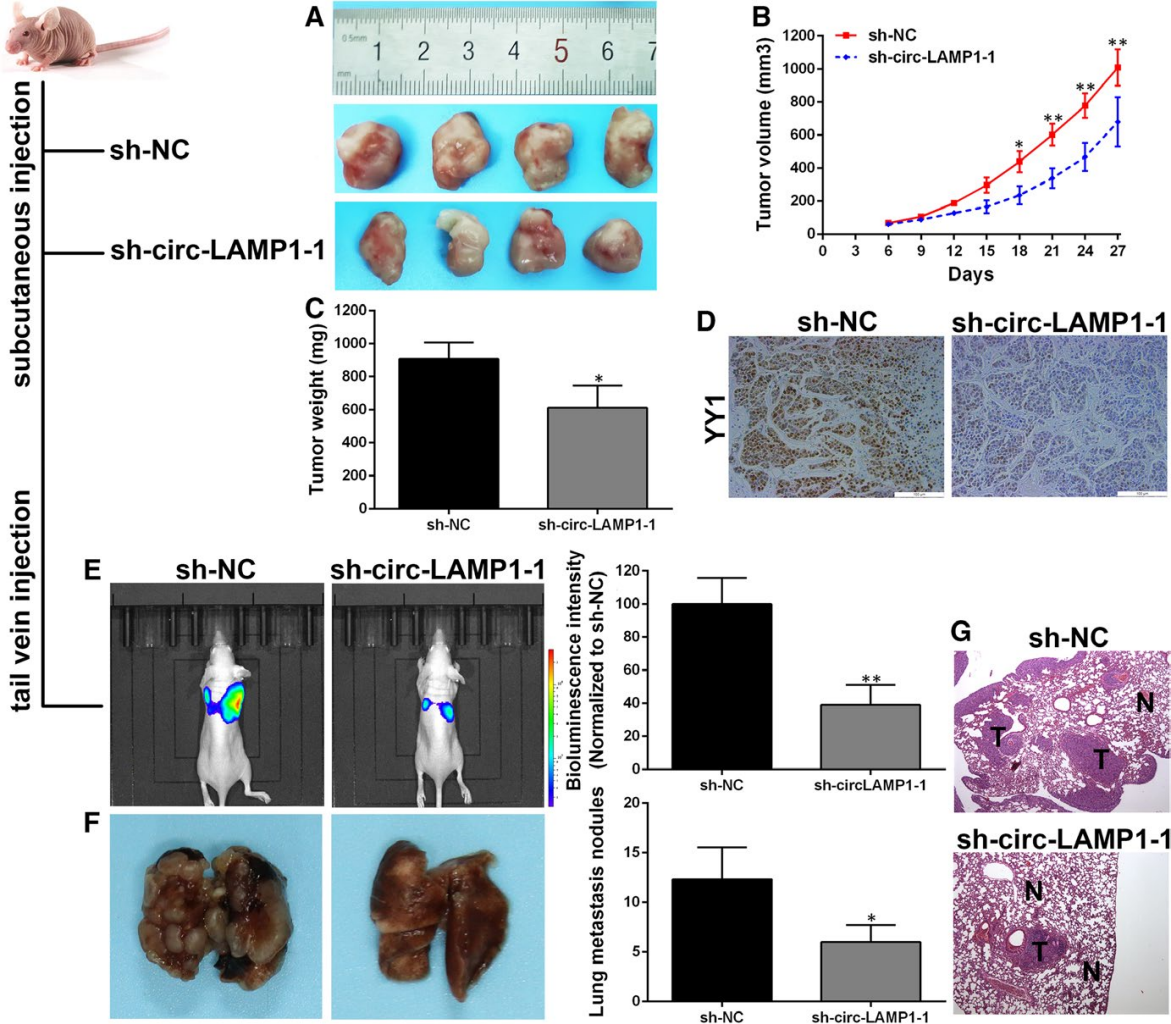
Circ‐LAMP1 promotes CCA cell growth and metastasis in vivo. A, The xenografts were removed at 27 days after injection. B, Growth curves of subcutaneous tumours. C, Tumours weight was measured. D, YY1 detection by IHC. E, Bioluminescence imaging of in vivo metastatic activity in each group. F, Statistical analysis of mice with lung metastasis in each group. G, H&E staining showing the metastatic tumours in lungs. *P < .05, **P < .01

In view of the foregoing description, we attempted to explore the role of circ‐LAMP1 in vivo. KMBC cells were transfected with sh‐circ‐LAMP1‐1. The transfected cells were inoculated into nude mice. As a result, the tumour volume (Figure 8A, B) and weight (Figure 8C) were sharply diminished via circ‐LAMP1 absence. Subsequently, we focused on the level of YY1 in xenograft tumours. Immunohistochemistry determined that YY1 was decreased in the tumours formed from circ‐LAMP1 depleted KMBC cells (Figure 8D). For lung metastasis model, a weaker fluorescence intensity of lungs was observed in the mice injected with circ‐LAMP1 decreased KMBC cells (Figure 6E). The lungs of the nude mice were harvested after four weeks inoculation. Decreased circ‐LAMP1 led to less lung metastases (Figure 6F, G).

## 4 DISCUSSION

Although therapy has been standardized, the rate of morbidity and mortality for CCA remains high at present.^15^ In recent years, the role of circRNAs in CCA already has made significant progress in basic medical research.^16^, ^17^ Recently, diagnosis and therapy from the perspective of circRNA has gradually become a new approach in cancer research. Therefore, exploring the CCA‐related circRNAs and find the ideal molecular markers for targeted therapy of CCA is the direction to further improve the prognosis of CCA.^18^, ^19^ Nevertheless, the functions and exact mechanisms of novel circRNAs in CCA are still not fully understood.

The current work identifies a novel circRNA, circ‐LAMP1, which is enhanced in CCA specimens screened by circRNA microarray. In the current work, we investigated its clinical significance and found the patients with high expression of circ‐LAMP1 was linked to more than one tumour, and higher TNM stages. Additionally, analysis data suggested that circ‐LAMP1 might be an independent biomarker for CCA prognosis/recurrence. Furthermore, in vitro and in vivo experiments revealed its effect in promoting the growth and metastasis of CCA cells, whereas we found circ‐LAMP1 expression did not affect the proliferation and apoptosis of normal biliary epithelium (HIBEC), indicating its limited side effect on normal cells.

CircRNA‐miRNA‐mRNA regulatory axis has been widely concerned on account of their rising role in human cancers.^20^, ^21^, ^22^ The tumour progression‐promoting effect of circ‐LAMP1 was also confirmed in T‐cell lymphoblastic lymphoma previously.^14^ Deng et al unveiled that circ‐LAMP1 contributes to cell proliferation through playing as a ceRNA for miR‐615‐5p to up‐regulate DDR2 level.^14^ However, we found miR‐615‐5p could not interacted with circ‐LAMP1 in our study, suggesting the tissue specific mechanism of circ‐LAMP1 in human cancers. In this study, up‐regulation of circ‐LAMP1 induced cell proliferation, metastasis and hampered cell apoptosis in CCA cells by absorbing miR‐556‐5p and miR‐567. MiR‐556‐5p is a newly identified tumour suppressive miRNA in human cancers. Recently, the study illustrated that miR‐556‐5p, negatively regulated by long non‐coding RNA SNHG1, suppressed meningioma progression through Wnt signalling pathway.^23^ In this work, it is the first time to demonstrate its tumour suppressing role in CCA. The role of miR‐567 in the facilitation or inhibition of tumour progression is still controversial. In osteosarcoma, miR‐567 decreases cell growth and metastatic properties through modulating FGF5.^24^ Similarly, decreased miR‐567 in breast cancer cells contributes to carcinogenesis.^25^ However, up‐regulation of circ‐cMras suppressed lung adenocarcinoma tumorigenesis by targeting miR‐567/PTPRG signalling, suggesting the oncogenic function of miR‐567 in lung adenocarcinoma.^26^ In the current study, the data agreed with the studies in osteosarcoma and breast cancer.^24^, ^25^

The downstream target of miR‐556‐5p and miR‐567 was further unveiled. YY1 belongs to the GLI‐Kruppel family which could activate or inactivate gene expression depending on interacting partners, promoter context and chromatin structure.^27^, ^28^ YY1 has been identified up‐regulated and functioned as an oncogene in several kind of cancers.^29^ Consistent with our expectation, we identified that the oncogenic properties induced by circ‐LAMP1 are partly dependent on its up‐regulation of YY1 in CCA. However, there are still several limitations within the current study. For example, the downstream target of YY1 was not evaluated and needs further study.

To sum up, the current study revealed that circ‐LAMP1 was overexpressed in CCA tissue specimens and cells. Circ‐LAMP1 functions as a ceRNA for miR‐556‐5p and miR‐567 to up‐regulate YY1 expression. The data clarified the potential mechanisms related to circ‐LAMP1 in regulating CCA cell growth and metastasis. This study suggests that circ‐LAMP1 might be a potential treatment target for CCA.

## 5 ETHICS APPROVAL AND CONSENT TO PARTICIPATE

This study was approved by the Ethics Committee of the Second Affiliated Hospital of Harbin Medical University.

## CONFLICT OF INTEREST

The authors declare that they have no competing interests regarding the publication of this paper.

## AUTHOR CONTRIBUTION

**Yi Xu:** Funding acquisition (equal); Investigation (equal); Methodology (equal). **Ping Gao:** Data curation (equal); Formal analysis (equal); Investigation (equal). **Zhidong Wang:** Formal analysis (equal); Investigation (equal); Software (equal); Visualization (equal); Writing‐original draft (equal). **Zhilei Su:** Investigation (equal); Validation (equal); Writing‐review & editing (equal). **Guanqun Liao:** Investigation (equal). **Yi Han:** Investigation (equal). **Yifeng Cui:** Data curation (equal); Writing‐review & editing (equal). **Yue Yao:** Conceptualization (equal); Funding acquisition (equal); Project administration (equal). **Xiangyu Zhong:** Conceptualization (equal); Supervision (equal).

## Supporting information

Table S1

Table S2

Table S3

## Data Availability

All data obtained during this research are available within the paper or available from the authors as needed.
