# Supplementary material for: Circ‐LAMP1 contributes to the growth and metastasis of cholangiocarcinoma via miR‐556‐5p and miR‐567 mediated YY1 activation
Source: J Cell Mol Med. 2021 Mar 6;25(7):3226–38. doi: 10.1111/jcmm.16392 (PMC8034453; doi:10.1111/jcmm.16392)
Supplement: Supplementary file 1 — Table S1 [file JCMM-25-3226-s002.docx]

**Table S1** Association between circ-LAMP1 expression and clinicopathological characteristics of CCA patients

| Clinicopathological | No. of | circ-LAMP1 expression | | *p*-value |
| --- | --- | --- | --- | --- |
| characteristics | patients | High (%) | Low (%) |  |
| Gender |  |  |  | 0.489 |
| Male | 128 | 67(31.02%) | 61(28.24%) |  |
| Female | 88 | 41(18.98%) | 47(21.76%) |  |
| Age (years) |  |  |  | 0.301 |
| <48 | 66 | 37(17.13%) | 29(13.43%) |  |
| ≥48 | 150 | 71(32.87%) | 79(36.57%) |  |
| Differentiation grade |  |  |  | 0.670 |
| Well/moderately | 76 | 40(18.52%) | 36(16.67%) |  |
| Poorly/undifferentiated | 140 | 68(31.48%) | 72(33.33%) |  |
| Tumor thrombus |  |  |  | 0.629 |
| Positive | 50 | 23(10.65%) | 27(12.50%) |  |
| Negative | 166 | 85(39.35%) | 81(37.50%) |  |
| Number of tumors |  |  |  | **0.003** |
| 1 | 140 | 59(27.31%) | 81(37.50%) |  |
| >1 | 76 | 49(22.69%) | 27(12.50%) |  |
| Tumor size |  |  |  | 0.413 |
| >5 cm | 99 | 53(24.54%) | 46(21.30%) |  |
| ≤5 cm | 117 | 55(25.46%) | 62(28.70%) |  |
| Lymph node metastasis |  |  |  | 0.076 |
| Positive | 39 | 25(11.57%) | 14(6.48%) |  |
| Negative | 177 | 83(38.43%) | 94(43.52%) |  |
| TNM stage |  |  |  | **0.013** |
| I-II | 146 | 64(29.63%) | 82(37.96%) |  |
| III-IV | 70 | 44(20.37%) | 26(12.04%) |  |
| Liver cirrhosis |  |  |  | 0.570 |
| Positive | 77 | 41(18.98%) | 36(16.67%) |  |
| Negative | 139 | 67(31.02%) | 72(33.33%) |  |
| HBV infection |  |  |  | 0.878 |
| Positive | 58 | 30(13.89%) | 28(12.96%) |  |
| Negative | 158 | 78(36.11%) | 80(37.04%) |  |
| Serum AFP |  |  |  | 0.706 |
| >25 ng/ml | 33 | 15(6.94%) | 18(8.33%) |  |
| ≤25 ng/ml | 183 | 93(43.06%) | 90(41.67%) |  |
| Serum CEA |  |  |  | 0.157 |
| >5 ng/ml | 54 | 32(14.81%) | 22(10.19%) |  |
| ≤5 ng/ml | 162 | 76(35.19%) | 86(39.81%) |  |
| Serum CA19-9 |  |  |  | 0.275 |
| >37 u/ml | 117 | 63(29.17%) | 54(25.00%) |  |
| ≤37 u/ml | 99 | 45(20.83%) | 54(25.00%) |  |

Data in bold indicates statistical significance at *p*<0.05.
