# Supplementary material for: Circ‐LAMP1 contributes to the growth and metastasis of cholangiocarcinoma via miR‐556‐5p and miR‐567 mediated YY1 activation
Source: J Cell Mol Med. 2021 Mar 6;25(7):3226–38. doi: 10.1111/jcmm.16392 (PMC8034453; doi:10.1111/jcmm.16392)
Supplement: Supplementary file 2 — Table S2 [file JCMM-25-3226-s001.docx]

**Table S2** Univariate and multivariate analysis of prognostic factors for overall survival in CCA patients

| Variables | Univariate analysis | | | Multivariate analysis | | |
| --- | --- | --- | --- | --- | --- | --- |
|  | HR | 95% CI | *p*-value | HR | 95% CI | *p*-value |
| Overall Survival | | | | | | |
| Gender  (Male vs. Female) | 1.479 | 1.007-2.174 | **0.046** | 1.409 | 0.951-2.088 | 0.088 |
| Age  (≥48 vs. <48) | 0.756 | 0.520-1.100 | 0.144 |  |  |  |
| Differentiation grade (Poorly/undifferentiated vs. Well/moderately) | 1.131 | 0.773-1.655 | 0.526 |  |  |  |
| Tumor thrombus  (Positive vs. Negative) | 1.474 | 0.966-2.250 | 0.072 |  |  |  |
| Number of tumors  (>1 vs. 1) | 2.039 | 1.409-2.949 | **<0.001** | 1.424 | 0.963-2.105 | 0.076 |
| Tumor size  (>5 cm vs. ≤5 cm) | 2.061 | 1.426-2.980 | **<0.001** | 1.738 | 1.163-2.595 | **0.007** |
| Lymph node metastasis (Positive vs. Negative) | 2.542 | 1.648-3.920 | **<0.001** | 1.685 | 0.919-3.090 | 0.092 |
| TNM stage  (III-IV vs. I-II) | 2.251 | 1.549-3.271 | **<0.001** | 1.248 | 0.727-2.142 | 0.422 |
| Liver cirrhosis  (Positive vs. Negative) | 0.819 | 0.556-1.206 | 0.312 |  |  |  |
| HBV infection  (Positive vs. Negative) | 1.150 | 0.774-1.710 | 0.489 |  |  |  |
| Serum AFP  (>25 ng/ml vs. ≤25 ng/ml) | 0.808 | 0.482-1.355 | 0.419 |  |  |  |
| Serum CEA  (>5 ng/ml vs. ≤5 ng/ml) | 2.045 | 1.361-3.073 | **0.001** | 1.607 | 1.036-2.493 | **0.034** |
| Serum CA19-9  (>37 u/ml vs. ≤37 u/ml) | 1.455 | 1.008-2.100 | **0.045** | 1.297 | 0.882-1.908 | 0.186 |
| Circ-LAMP1 expression  (High vs. Low) | 2.300 | 1.588-3.332 | **<0.001** | 1.906 | 1.288-2.821 | **0.001** |

HR: hazard ratio, 95 % CI: 95 % confidence interval

Data in bold indicates statistical significance at *p*<0.05.
