# Supplementary material for: Circ‐LAMP1 contributes to the growth and metastasis of cholangiocarcinoma via miR‐556‐5p and miR‐567 mediated YY1 activation
Source: J Cell Mol Med. 2021 Mar 6;25(7):3226–38. doi: 10.1111/jcmm.16392 (PMC8034453; doi:10.1111/jcmm.16392)
Supplement: Supplementary file 3 — Table S3 [file JCMM-25-3226-s003.docx]

**Table S3** Univariate and multivariate analysis of prognostic factors for disease-free survival in CCA patients

| Variables | Univariate analysis | | | Multivariate analysis | | |
| --- | --- | --- | --- | --- | --- | --- |
|  | HR | 95% CI | *p*-value | HR | 95% CI | *p*-value |
| Disease-free Survival | | | | | | |
| Gender  (Male vs. Female) | 1.421 | 1.002-2.014 | **0.049** | 1.383 | 0.967-1.979 | 0.075 |
| Age  (≥48 vs. <48) | 0.833 | 0.588-1.181 | 0.305 |  |  |  |
| Differentiation grade (Poorly/undifferentiated vs. Well/moderately) | 1.103 | 0.780-1.559 | 0.580 |  |  |  |
| Tumor thrombus  (Positive vs. Negative) | 1.197 | 0.802-1.787 | 0.379 |  |  |  |
| Number of tumors  (>1 vs. 1) | 2.101 | 1.495-2.951 | **<0.001** | 1.645 | 1.157-2.340 | **0.006** |
| Tumor size  (>5 cm vs. ≤5 cm) | 1.879 | 1.346-2.623 | **<0.001** | 1.679 | 1.171-2.407 | **0.005** |
| Lymph node metastasis (Positive vs. Negative) | 2.574 | 1.722-3.848 | **<0.001** | 1.636 | 0.917-2.916 | 0.095 |
| TNM stage  (III-IV vs. I-II) | 2.045 | 1.450-2.885 | **<0.001** | 1.217 | 0.737-2.011 | 0.443 |
| Liver cirrhosis  (Positive vs. Negative) | 0.939 | 0.663-1.332 | 0.726 |  |  |  |
| HBV infection  (Positive vs. Negative) | 1.299 | 0.910-1.856 | 0.150 |  |  |  |
| Serum AFP  (>25 ng/ml vs. ≤25 ng/ml) | 0.940 | 0.589-1.498 | 0.793 |  |  |  |
| Serum CEA  (>5 ng/ml vs. ≤5 ng/ml) | 1.721 | 1.183-2.504 | **0.005** | 1.346 | 0.903-2.005 | 0.144 |
| Serum CA19-9  (>37 u/ml vs. ≤37 u/ml) | 1.410 | 1.010-1.967 | **0.043** | 1.300 | 0.919-1.838 | 0.138 |
| Circ-LAMP1 expression  (High vs. Low) | 2.257 | 1.609-3.166 | **<0.001** | 1.817 | 1.272-2.595 | **0.001** |

Data in bold indicates statistical significance at *p*<0.05.
